# Activation of Tenofovir Alafenamide and Sofosbuvir in the Human Lung and Its Implications in the Development of Nucleoside/Nucleotide Prodrugs for Treating SARS-CoV-2 Pulmonary Infection

**DOI:** 10.3390/pharmaceutics13101656

**Published:** 2021-10-11

**Authors:** Jiapeng Li, Shuhan Liu, Jian Shi, Hao-Jie Zhu

**Affiliations:** Department of Clinical Pharmacy, College of Pharmacy, University of Michigan, Ann Arbor, MI 48109, USA; ljiapeng@med.umich.edu (J.L.); shuliu@med.umich.edu (S.L.); jianshi@umich.edu (J.S.)

**Keywords:** tenofovir alafenamide, sofosbuvir, prodrug, COVID-19, cathepsin A, carboxylesterase 1

## Abstract

ProTide technology is a powerful tool for the design of nucleoside/nucleotide analog prodrugs. ProTide prodrug design improves cell permeability and enhances intracellular activation. The hydrolysis of the ester bond of a ProTide is a determinant of the intracellular activation efficiency and final antiviral efficacy of the prodrug. The hydrolysis is dictated by the catalytic activity and abundance of activating enzymes. The antiviral agents tenofovir alafenamide (TAF) and sofosbuvir (SBV) are typical ProTides. Both TAF and SBV have also been proposed to treat patients with COVID-19. However, the mechanisms underlying the activation of the two prodrugs in the lung remain inconclusive. In the present study, we profiled the catalytic activity of serine hydrolases in human lung S9 fractions using an activity-based protein profiling assay. We evaluated the hydrolysis of TAF and SBV using human lung and liver S9 fractions and purified enzymes. The results showed that CatA and CES1 were involved in the hydrolysis of the two prodrugs in the human lung. More specifically, CatA exhibited a nearly 4-fold higher hydrolytic activity towards TAF than SBV, whereas the CES1 activity on hydrolyzing TAF was slightly lower than that for SBV. Overall, TAF had a nearly 4-fold higher hydrolysis rate in human lung S9 than SBV. We further analyzed protein expression levels of CatA and CES1 in the human lung, liver, and primary cells of the two tissues using proteomics data extracted from the literature. The relative protein abundance of CatA to CES1 was considerably higher in the human lung and primary human airway epithelial cells than in the human liver and primary human hepatocytes. The findings demonstrated that the high susceptivity of TAF to CatA-mediated hydrolysis resulted in efficient TAF hydrolysis in the human lung, suggesting that CatA could be utilized as a target activating enzyme when designing antiviral ester prodrugs for the treatment of respiratory virus infection.

## 1. Introduction

**Figure 1:**
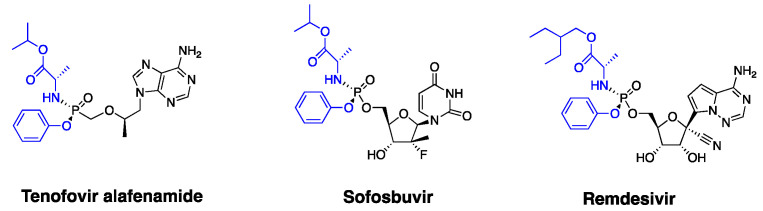
Chemical structures of the three FDA-approved antiviral ProTides, tenofovir alafenamide (TAF), sofosbuvir (SBV), and remdesivir (RDV). Two oxygens of monophosphate/monophosphonate are masked by an aryloxy group and an amino acid ester group (highlighted in blue).

Nucleoside/nucleotide analogs remain an important class of antivirals in clinics. These therapeutics commonly exert their antiviral effects intracellularly through the formed active metabolites, triple phosphate nucleoside (TP-Nuc), which inhibit the viral polymerase or/and terminate the elongation of the viral nucleic acid chain [1]. However, nucleotide/nucleoside analogs have limitations, such as limited cell permeability associated with the polar molecular structure and inefficient intracellular activation. To improve cell permeability and pharmacokinetic performance, nucleoside/nucleotide analogs are often structurally modified to be ester prodrugs [2]. The ProTide technology has been a powerful tool to improve the cell permeability and the intracellular activation of nucleoside/nucleotide antivirals and anticancer drugs [3,4]. The most recent ProTide technology is to structurally modify nucleoside/nucleotide analogs to aryloxy phosphoramidite prodrugs by masking two of the oxygens of the monophosphate and monophosphonate groups with an aryloxy group and an amino acid ester group. The ProTide prodrugs have two advantages over parent nucleoside/nucleotide forms: (1) great cell permeability due to low polarity; (2) rapid intracellular activation by avoiding the rate-limiting monophosphorylating process [3,5,6,7]. There are at least three FDA-approved ProTide prodrugs, including tenofovir alafenamide (TAF), sofosbuvir (SBV), and remdesivir (RDV) (Figure 1). Among these, TAF has been commonly used against hepatitis B virus (HBV) and human immunodeficiency virus (HIV), while SBV is mainly used for treating hepatitis C virus (HCV) infection. RDV was recently approved for the treatment of coronavirus disease 2019 (COVID-19) due to its promising antiviral effects against severe acute respiratory syndrome coronavirus-2 (SARS-CoV-2). TAF and SBV have also been proposed to be alternatives for COVID-19 treatment based on their anti-SARS-CoV-2 effects observed in vitro [8,9].

**Figure 2:**
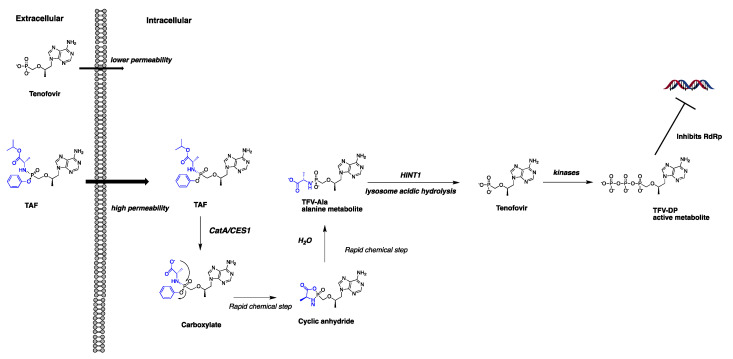
The putative intracellular activation pathway of TAF. TAF exhibits a significantly improved cell permeability compared to tenofovir. After cell entry, TAF is hydrolyzed by CatA and CES1 and is converted to its alanine metabolite (TFV-Ala) following several rapid chemical reaction steps. TFV-Ala is further hydrolyzed by HINT1 to form the monophosphonate product, tenofovir, which is subsequently phosphorylated by kinases to generate the active metabolite tenofivir-diphosphote (TFV-DP).

Besides the antiviral potency observed in vitro, whether the in situ intracellular concentration of active metabolite reaches and maintains the effective level (e.g., EC_50_) is also critical for the successful treatment of an antiviral prodrug. It has been well documented that TAF [10,11], SBV [12], and RDV [13] undergo a similar metabolism pathway mediated by a series of enzymes to generate the active metabolite, TP-Nuc. After entering cells, the ester groups are reportedly cleaved by cathepsin A (CatA) and carboxylesterase 1 (CES1), and then the P-N bond is cleaved mainly by histidine triad nucleotide-binding protein 1 (HINT1) to release the MP-Nuc metabolite. The MP-Nuc is subsequently phosphorylated by kinases to form TP-Nuc [12,13,14] (Figure 2, a putative metabolism pathway exemplified by the TAF metabolism). Multiple studies have shown that the antiviral efficacy of TAF, SBV, and RDV is highly associated with the ester hydrolysis rate, and the hydrolysis process is dependent on the activity and the protein abundance of CatA and CES1 [12,13,14]. Recently, some scholars indicated that the ProTide technology might be more suitable for therapeutics against liver diseases (e.g., HBV and HCV) rather than lung diseases because a large fraction of the prodrug would be hydrolyzed and exert ensuing antiviral efficacy in the liver due to the high abundance of CES1 and CatA in the liver [15]. A more recent study, however, demonstrated that human lung tissues also have sufficient CatA and CES1 to activate RDV [13]. Our recent study found that CES1 protein abundance was over 200-fold higher in the liver than in the lung, while CatA protein expression was comparable between the lung and the liver [16]. As such, we hypothesized that a greater susceptivity to CatA and lower susceptivity to CES1 might facilitate the pulmonary activation of a ProTide. The activity of CatA for TAF hydrolysis has been reported to be higher than that for SBV and RDV [12,13,14]. However, to our knowledge, the hydrolysis profiles of TAF and SBV in human lungs have not yet been evaluated.

Serine hydrolases, including CES1, CatA, and more than 200 other members, are among the largest and most diverse enzyme classes in humans [17]. Some of the enzymes catalyze ester hydrolysis and are involved in nucleoside/nucleotide ester prodrug hydrolysis [2]. Most of these enzymes exert their catalytic functions via nucleophilic attacking the carbonyl carbon atom of the carboxylic ester through its serine-histidine-aspartate triad [18]. Activity-based protein profiling (ABPP) is a powerful chemoproteomic strategy for assessing the catalytic activity of target enzymes in native biological systems [17,18,19,20]. In this study, we conducted an ABPP study using the desthiobiotin-fluorophosphonate (FP) probe [18] to globally assess the catalytic activity of serine hydrolases in human lung S9 (HlungS9) fractions in an effort to identify hydrolyses that can be targeted for activating an ester prodrug in the human lung. We further investigated the hydrolysis of TAF and SBV in HlungS9 and by purified CatA and CES1 to assess whether TAF and SBV could be effective oral drugs to treat human respiratory infectious diseases (e.g., COVID-19) from a prodrug activation perspective.

## 2. Materials and Methods

### 2.1. Materials

TAF, SBV, RDV, and telaprevir were purchased from MedChemExpress (Monmouth Junction, NJ, USA). Recombinant human CatA (rhCatA), CathepsinL (rhCatL), and CES1 (rhCES1) were purchased from R&D Systems (Minneapolis, MN, USA). Recombinant human lysophospholipase 1 (rhLYPLA1) was purchased from OriGene (Rockville, MD, USA). Pooled human liver S9 fraction (HLS9) and pooled HlungS9 were products from XenoTech LLC (Kansas City, KS, USA). The demographic information about the tissue donors is summarized in Table S1. The ActivX™ desthiobiotin-FP probe and Pierce™ streptavidin agarose resin were obtained from Thermo Scientific (Rockford, IL, USA). Bis-(p-nitrophenyl) phosphate (BNPP), dithiolthreitol, iodoacetamide, urea, ammonium bicarbonate, and Tris-base (Trizma base) were purchased from Sigma-Aldrich (St. Louis, MO, USA). Tris-HCl was purchased from Fisher Scientific (Fair Lawn, NJ, USA). The MES buffer (0.2 M, pH 5.5) was purchased from Alfa Aesar (Ward Hill, MA, USA). All other chemicals and reagents were of analytical grade and commercially available.

### 2.2. ABPP of Serine Hydrolases HlungS9

The experimental method was based on a previous ABPP proteomics study with some modifications [21]. The mechanism of this ABPP method is summarized in Figure S1. Briefly, 200 μg HlungS9 protein was diluted with PBS to 1 μg/μL. The protein solution (200 μL) was incubated with 2 μM (final concentration) ActivX™ desthiobiotin-FP probe or vehicle (DMSO, final concentration: 1%, *v*/*v*) for 1 and 12 min at 37 °C on a shaker (Benchmark, Multi-Therm^TM^, Tempe, AZ, USA) at 1200 rpm. The labeling reaction was terminated by adding 1.6 mL of acetone containing the internal standard (IS) biotinylated BSA (600 ng). The mixture was vortexed for 1 min and stored at 4 °C overnight before being centrifuged at 21,130× *g* for 15 min to remove the supernatant. The excess probe was removed by washing twice with 1 mL methanol. The precipitated proteins were resuspended in 200 μL 8 M Urea/100 mM ammonium bicarbonate solution containing 4 mM dithiolthreitol (DTT), incubated at 37 °C for 45 min, and shaken at 1200 rpm. The sample was allowed to cool to room temperature before adding 40 μL (0.5 M) iodoacetamide (IAA). The mixture was placed in the dark for 30 min for alkylation. The sample was then diluted with 1.6 mL of PBS. Then, 50 μL of 50% slurry of Pierce™ streptavidin agarose (beads) was washed with PBS (20 mL) three times and mixed with the proteome sample. The mixture was incubated for 3 h at room temperature with gentle shaking on a shaker (Benchmark, B3D 1320 Super Nutation Mixer). The beads were then isolated by centrifugation and washed five times with 0.5 mL of 0.8 M urea and then five times with 0.5 mL of PBS. The proteins on the beads were digested overnight with sequencing grade trypsin in 500 μL PBS at 37 °C with gentle shaking. The digested peptides were extracted and cleaned using Waters Oasis HLB columns following the manufacturer’s instructions. The eluted peptides were dried and stored at −80 °C for liquid chromatography–tandem mass spectrometry (LC-MS/MS) analysis.

### 2.3. Enzyme and Tissue S9 Activity Assays

The activity of rhCatA and rhCES1 for hydrolyzing TAF and SBV was measured using a method reported in a previous study [12] with minor modifications. Briefly, the optimized buffer systems for rhCatA and rhCES1 were MES assay buffer (pH 5.5, 0.2 M MES buffer containing 100 mM NaCl, 1 mM DTT, and 0.1% Nonidet) and Tris buffer (pH 7.5, 50 mM Tris, prepared by dissolving 6.06 g Tris-HCL and 1.39 g Tris-base in 1000 mL water), respectively. rhCatA was activated by rhCatL following the manufacturer’s instructions. The CatA assay was carried out in a 50 µL reaction mixture containing 0.5 ng/µL activated rhCatA and 20 µM TAF or 20 µM SBV in the MES assay buffer (pH 5.5). The rhCatA assay mixture was incubated at 37 °C for 5 min with shaking at 1200 rpm. The rhCES1 assay was conducted in a 20 µL reaction mixture containing 40 ng/µL rhCES1 and 20 µM TAF or 20 µM SBV in the Tris buffer (pH 7.5). The rhCES1 assay mixture was incubated at 37 °C for 20 min with shaking at 1200 rpm. Our preliminary study confirmed a linear relationship of TAF hydrolysis with the tested enzyme concentrations (CatA: 0.1–0.5 ng/µL; CES1: 20–40 ng/µL) and the incubation times (CatA: 0–5 min; CES1: 0–30 min) and a linear relationship of SBV hydrolysis with CES1 concentrations (20–40 ng/µL) and incubation times (CES1: 0–40 min). The tested SBV concentration (20 µM) was well below the K_m_ value (700 µM) for CatA reported in a previous study [12]. The rhLYPLA1 activity for TAF hydrolysis was tested in Tris pH 7.5 condition because its subcellular expression location is mainly cytosol [22]. The rhLYPLA1 assay was conducted in a 50 µL reaction mixture containing 20 or 40 ng/µL rhLYPLA1 and 20 µM TAF at 37 °C for 15 and 30 min with shaking at 1200 rpm on a shaker (Benchmark, Multi-Therm^TM^). The enzyme activity was calculated by dividing the reduction of TAF or SBV by the enzyme concentration and incubation duration time.

The tissue S9 assay was carried out in MES assay buffer (pH 5.5) and Tris buffer (pH 7.5) to optimize the activity of native CatA and CES1, respectively. Then, 20 µM prodrug (TAF or SBV) was incubated with 0.5 mg/mL HLungS9 or HLS9 protein at 37 °C for 15 min and 30 min with shaking at 1200 rpm on a shaker (Benchmark, Multi-Therm^TM^). The reactions were terminated by adding a two-fold volume of acetonitrile (ACN) containing the internal standard RDV (5 µM). The samples were then vortexed for 0.5 min and centrifuged at 21,130× *g* for 10 min at 4 °C to remove the precipitated proteins. The resulting supernatant was collected and diluted with ten volumes of 3% (*v*/*v*) ACN containing 0.1% (*v*/*v*) formic acid before being injected into an LC-MS/MS system with an assay method described below.

### 2.4. Inhibition Assay

An in vitro study was performed to evaluate the effects of the CatA inhibitor telaprevir and the CES1 inhibitor BNPP on TAF hydrolysis in HlungS9. TAF concentration was determined after incubation of TAF (20 µM) with pooled HlungS9 (0.5 mg/mL) at 37 °C for 30 min in the absence or presence of various concentrations of telaprevir (0.5, 5, and 50 µM) and BNPP (1, 10, and 50 µM). The reactions were terminated by adding a two-fold volume of ACN containing the internal standard RDV (5 µM). The samples were then vortexed for 1 min and centrifuged at 21,130× *g* for 10 min at 4 °C to remove the precipitated proteins. The resulting supernatant was collected to analyze TAF using an LC-MS/MS assay described below.

### 2.5. LC-MS/MS-Based Proteomics Analysis

We used an established LC-MS/MS-based data-independent acquisition (DIA) proteomics approach [23,24] to quantify the relative abundance of serine hydrolases enriched by ActivX™ desthiobiotin-FP probe from HlungS9 in the ABPP experiment. The analysis was carried out on a TripleTOF 5600 plus mass spectrometer (AB Sciex, Framingham, MA, USA) coupled with an Eksigent 2D plus LC system (Eksigent Technologies, Dublin, CA, USA). Briefly, we used a trapping column (ChromXP C18-CL, 120 Å, 5 mm, 0.3 mm cartridge; Eksigent Technologies) to load the samples and an analytical column (ChromXP C18-CL, 120 Å, 150 × 0.3 mm^2^, 5 mm; Eksigent Technologies) to separate peptides. The mobile phase consisted of water with 0.1% formic acid (A) and acetonitrile (ACN) containing 0.1% formic acid (B). Mobile phase A was delivered at a flow rate of 10 μL/min for 3 min to load 1~2 µg proteins to the trapping column. A gradient elution at a flow rate of 5 μL/min was used to separate the injected peptides on the analytical column. The gradient parameters are summarized in Table S2. A blank sample (30% ACN, *v*/*v*) was injected between each run to minimize carryover. MS data were collected in a positive mode with the source temperature set at 280 °C and the ion spray voltage of 3000 V for ionization.

### 2.6. LC-MS/MS Analysis of TAF and SBV

TAF and SBV were quantified based on a previously reported method with some modifications [25]. The LC-MS/MS system consisted of a PE SCIEX API 3000 spectrometer and a Shimadzu UFLC system (Shimadzu, Tokyo, Japan). We used a RESTEK Ultra II C18 column (5 μm, 50 cm × 2.1 mm, Bellefonte, PA, USA) with a RESTEK UltraShield UHPLC precolumn filter (0.2 μm frit, Bellefonte, PA, USA) for chromatographic separation at 45 °C. Mobile phase A was water containing 0.1% (*v*/*v*) formic acid, and mobile phase B was ACN containing 0.1% (*v*/*v*) formic acid. The mobile phase flow rate was 0.5 mL/min, and 1 μL sample was injected for analysis. For the gradient program, mobile phase B was at 5% for the first 0.1 min, increased to 90% during the period of 0.1 min–2 min and maintained at 90% for 1 min, then returned to 5% at 3.1 min and maintained at 5% until the end of the gradient at 5 min. We operated the MS in a positive mode with turbo electrospray ionization. We set the following MS parameters: nebulizer gas: 15 psi; curtain gas: 15 psi; collision gas: 6 psi; ionspray voltage: 5500 V; source temperature: 550 °C; declustering potential: 14 V; focusing potential: 30 V; entrance potential: 6 V; collision energy: 35 V; collision cell exit potential: 15 V. In a multiple reaction monitoring (MRM) mode, the following transitions were monitored: TAF, *m*/*z* 477.20 > 176.10; SBV, *m*/*z* 530.30 > 243.00; RDV, *m*/*z* 603.23 > 200.10. The peak area ratios of TAF and SBV to RDV were used to quantify TAF and SBV. The regression coefficients of calibration curves were greater than 0.99 analytes’ concentrations between 1.25 μM and 20 μM. Accuracy and precision results met the FDA bioanalytical method validation guidance requirements.

### 2.7. Meta-Analysis of CatA, CES1, HINT1 Protein Expression in Human Respiratory and Hepatic Systems

The protein expression data of CatA, CES1, and HINT1 in human respiratory (lungs and airways) and hepatic systems were retrieved from the literature. The protein expression data in HLS9 and HLungS9 were obtained from two recent studies [13,16]. The protein expression data of autopsy samples from COVID-19 patients’ lung and liver tissues were extracted from a recent study by Nie et al. [26]. The global proteome data of primary human tracheal bronchial epithelial cells (TBECs) from healthy nonsmokers (n = 4; males) were obtained from the study by Foster et al. [27], in which a label-free data-dependent acquisition (DDA) method was used for proteomics analysis, and the peptides raw data were provided [27]. We calculated the abundance of proteins of interest using a label-free DDA-total protein approach algorithm (DDA-TPA) established in our lab [23]. The proteome data of human primary hepatocytes from normal liver tissues of seven donors were obtained from the study by Wiśniewski et al. [28]. The protein expression data in Huh7 and A549 cell lines were extracted from a recent study by Nusinow et al. [29].

### 2.8. Data Analysis

Proteomics data were analyzed using the Spectronaut Pulsar software (version 11.0; Biognosys AG, Schlieren, Switzerland) with a spectral library generated from pooled HLS9 and HlungS9 in a previous study [16] and the human proteome FASTA file downloaded from UniProtKB. The human serine hydrolases proteomics data were extracted using a FASTA file containing 238 human serine hydrolases [16]. The normalized abundance of a specific serine hydrolase was the ratio of its intensity to the intensity of biotinylated BSA (internal standard) in each sample. The enrichment ratio of each hydrolase was calculated by dividing its normalized abundance in the probe-treated group with its normalized abundance in the control group.

The estimated contribution of CatA and CES1 to TAF and SBV hydrolysis was calculated by timing the activity of rhCatA and rhCES1 with the enzyme abundance in a specific biological system (e.g., HlungS9, HLS9, and primary human cells) [16]. It was assumed that the activity of a recombinant human enzyme was similar to the activity of its native counterpart in human tissue S9 and primary human cells. The total hydrolysis rate of TAF or SBV in a specific biological system was established by summing CatA and CES1 contributions.

GraphPad Prism version 8.3.0 (GraphPad Software, San Diego, CA, USA) was used for statistical analysis and generating graphs. The Student’s *t*-test was used to analyze the differences between the enzyme’s activity for TAF and SBV, and the difference between the remaining TAF and SBV after incubation with tissue S9 fractions. The analysis of variance (ANOVA) was sued to analyze the difference between groups treated by various concentrations of inhibitors and the difference of the estimated prodrug hydrolysis rates among the three human primary cells. A *p*-value less than 0.05 was considered statistically significant.

## 3. Results

### 3.1. Serine Hydrolase Activity Profiles in HlungS9

**Figure 3:**
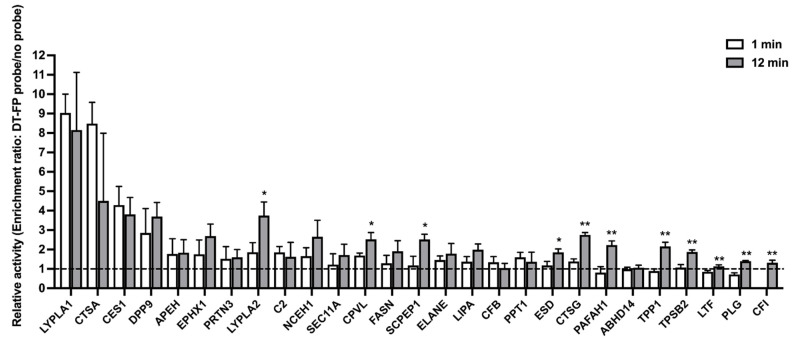
The relative catalytic activity of serine hydrolases in HlungS9. The relative activity was profiled by the enrichment ratio after 1-min and 12-min incubations with the desthiobiotin-FP probe. The enrich ratio of a given hydrolase was calculated by dividing its abundance in the probe treated samples by its abundance in the control samples (without a probe, 1% DMSO). Bars show mean values (±S.D.) of three independent experiments (n = 3), except for LYPLA2 in the 1-min enrichment profile (n = 2). Values below the dashed line (enrichment ratio = 1) indicate no enrichment. * *p* < 0.05 and ** *p* < 0.01 indicate statistically significant difference compared with the 1-min incubations (Student’s *t*-test).

The HlungS9 serine hydrolases ABPP results are shown in Figure 3. The desthiobiotin-FP probe enriched about 5 and 20 serine hydrolases after 1 min and 12 min probe labeling incubations, respectively. Specifically, LYPLA1 and CatA (CTSA) were enriched considerably faster than other enzymes by the desthiobiotin-FP probe. Moreover, LYPLA1, CatA, and CES1 showed the highest catalytic activity in both 1-min and 12-min incubations, indicating that the three enzymes are the predominant hydrolases in the human lung. No significant differences were observed in the enrichment folds of LYPLA1, CatA, and CES1 between 12-min and 1-min incubations, suggesting the enzymes could be rapidly enriched by the probe within 1 min. In comparison, the enrichment of several other serine hydrolases, including LYPLA2, CPVL, SCPEP1, ESD, CTSG, PAFAH1, TPP1, TPSB2, LTF, PLG, and CFI, was significantly increased by a longer period time of (12 min) probe incubation.

### 3.2. Enzyme Activity

**Figure 4:**
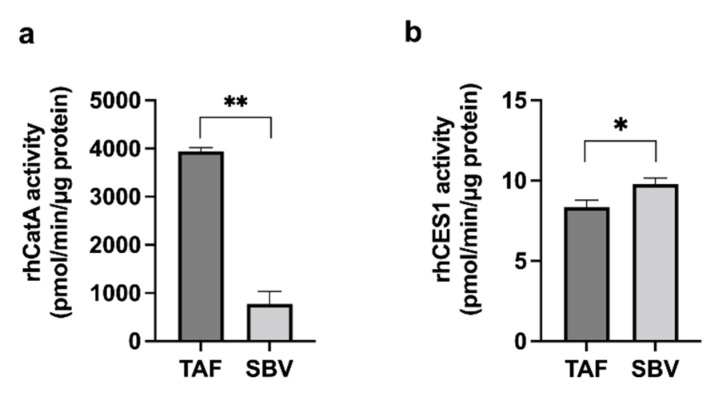
Activity of rhCatA (**a**) and rhCES1 (**b**) for TAF and SBV hydrolysis. 0.5 ng/µL rhCatA and 40 ng/µL rhCES1 were incubated with 20 µM TAF or SBV at 37 °C for 5 and 20 min, respectively. The enzyme activity was calculated by dividing the disappearance amount of the prodrug by the enzyme concentration and incubation time. Bars show mean values (±S.D.) of three independent experiments (n = 3). * *p* < 0.05 and ** *p* < 0.01 indicate statistically significant difference between TAF and SBV groups by Student’s *t*-test.

Based on the HlungS9 serine hydrolases ABPP result, we tested the activity of rhLYPLA1, rhCatA, and rhCES1 on hydrolyzing TAF. Both rhCatA and rhCES1 demonstrated significant activity; however, rhLYPLA1 showed no activity towards TAF hydrolysis even at high concentrations (20 and 40 ng/µL) (Figure S2). The activities of rhCatA and rhCES1 on SBV hydrolysis were also determined and compared with those for TAF (Figure 4). The activity of CatA for TAF was approximately 4-fold higher than that for SBV (3941 ± 77 vs. 772 ± 262 pmol/min/µg protein, n = 3, *p* < 0.01); while the CES1 activity for TAF was slightly lower than that for SBV (8.35 ± 0.43 vs. 9.79 ± 0.36 pmol/min/µg protein, n = 3, *p* < 0.01). The activity ratios of CatA to CES1 on hydrolyzing TAF and SBV were 472:1 and 79:1 for TAF and SBV, respectively, suggesting that, relative to SBV, TAF is more susceptible to CatA.

### 3.3. TAF and SBV Hydrolysis in HlungS9 and HLS9

**Figure 5:**
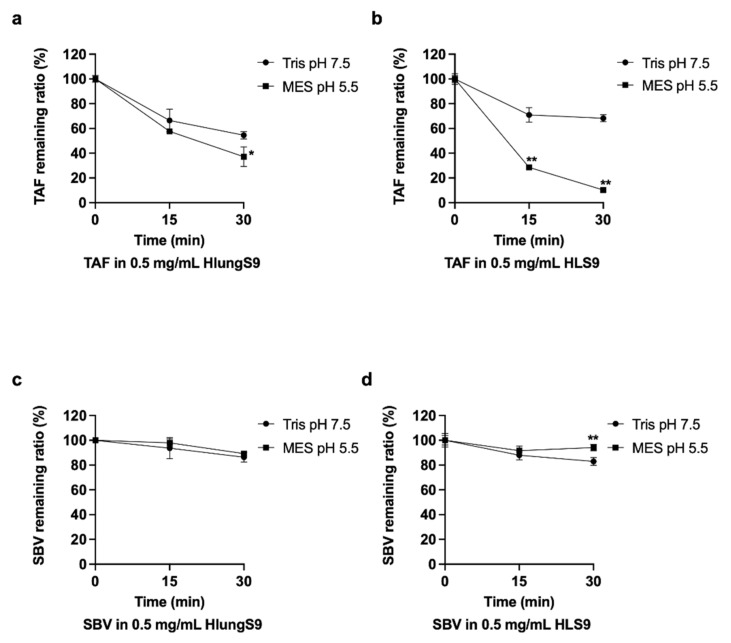
TAF and SBV hydrolysis in HlungS9 and HLS9: 20 µM TAF were incubated with 0.5 mg/mL HlungS9 (**a**) and HLS9 (**b**) at 37 °C for 15 and 30 min in MES assay buffer (pH 5.5) and Tris buffer (pH 7.5); 20 µM SBV were incubated with 0.5 mg/mL HlungS9 (**c**) and HLS9 (**d**) at 37 °C for 15 and 30 min in MES assay buffer (pH 5.5) and Tris buffer (pH 7.5). Data are shown as the remaining TAF or SBV (%) after incubations (mean ± S.D., n = 3, except for SBV with 0.5 mg/mL HlungS9 in MES pH 5.5, where n = 2). * *p* < 0.05 and ** *p* < 0.01 vs. the Tris pH 7.5 condition (Student’s *t*-test).

The hydrolysis of TAF and SBV in HlungS9 and HLS9 were determined (Figure 5). To optimize the performance of native CatA and CES1 in tissue S9, different pH buffer systems were adopted: MES pH 5.5 was the optimized condition for CatA, and Tris pH 7.5 was used for CES1. TAF showed a much more rapid hydrolysis rate than SBV in both HlungS9 and HLS9. Specifically, in HlungS9, TAF hydrolysis rate was approximately 4-fold higher than that of SBV. After 30 min incubations, about 65% and 45% TAF was hydrolyzed by HlungS9 in MES pH 5.5 and Tris pH 7.5, respectively. In comparison, only 15% and 10% SBV was hydrolyzed by HlungS9 in the pH 5.5 and pH 7.5 conditions, respectively. Both HlungS9 and HLS9 showed an enhanced TAF hydrolysis capability in pH 5.5 than in pH 7.5. However, the pH 5.5 condition did not significantly increase SBV hydrolysis in HlungS9 and HLS9.

### 3.4. CatA and CES1 Inhibitor Effects on TAF Hydrolysis in HlungS9

**Figure 6:**
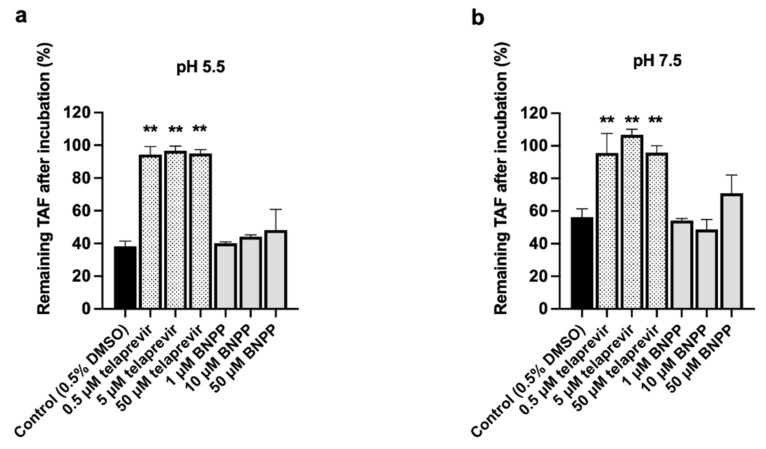
Effects of telaprevir and BNPP on TAF hydrolysis in HlungS9. TAF (20 µM) was incubated with 0.5 mg/mL of HlungS9 at 37 °C for 30 min with the presence of various concentrations of telaprevir (0.5, 5, and 50 µM) and BNPP (1, 10, and 50 µM). Incubations were performed in MES assay buffer (pH 5.5) (**a**) and Tris buffer (pH 7.5) (**b**) to optimize the activity of native CatA and CES1, respectively. Data are shown as the remaining TAF (%) after incubations (mean ± S.D., three independent experiments). ** *p* < 0.01 indicates statistically significant difference compared to the control group by ANOVA test.

To further confirm the role of CatA and CES1 in HlungS9 for TAF hydrolysis, we evaluated the effects of CatA inhibitor (telaprevir) and CES1 inhibitor (BNPP) on TAF hydrolysis in HlungS9. The inhibitory effects are displayed in Figure 6. In both the pH 5.5 and pH 7.5 conditions, telaprevir at 0.5~50 µM could nearly abolish TAF hydrolysis in HlungS9 (*p* < 0.01, compared with the control group). BNPP appeared to have a slight inhibitory effect on TAF hydrolysis in HlungS9 only at 50 µM without reaching a statistically significant difference (*p* = 0.15 and 0.09 compared with the control group at pH 5.5 and pH 7.5, respectively).

### 3.5. Meta-Analysis of the Abundance of CatA and CES1 in Human Respiratory and Hepatic Systems

The abundance of CatA and CES1 in human lung and liver tissues and primary cells are summarized in Table 1. CatA abundance in HlungS9 was comparable to that in HLS9, and CatA abundance in primary human airway epithelial cells was comparable to that in primary human hepatocytes. Unlike CatA, the abundance of CES1 was much higher in HLS9 and primary human hepatocytes than in HlungS9 and primary human airway epithelial cells. The abundance ratio of CatA to CES1 in HlungS9 and primary human airway epithelial cells was much higher than that in HLS9 and primary hepatocytes, respectively (Table 1). Based on the enzymatic activity and protein abundance, CatA was estimated to account for 97~99% of TAF hydrolysis in HlungS9 and primary human airway epithelial cells. In HLS9 and primary human hepatocytes, CatA contributes to TAF hydrolysis to a less extent (64~76%). The abundance of HINT1, the enzyme for converting TAF ester hydrolysis product to the monophosphate nucleoside (tenofovir), is summarized in Table S3. HLS9 had a relatively higher abundance of HINT1 than HlungS9, while primary human airway epithelial cells expressed relatively more HINT1 than primary human hepatocytes. Nevertheless, the differences of CatA and HINT1 expression between the human lung and liver tissues and cells were generally much less than the tissue/cell differences in CES1 expression.

**Figure 7:**
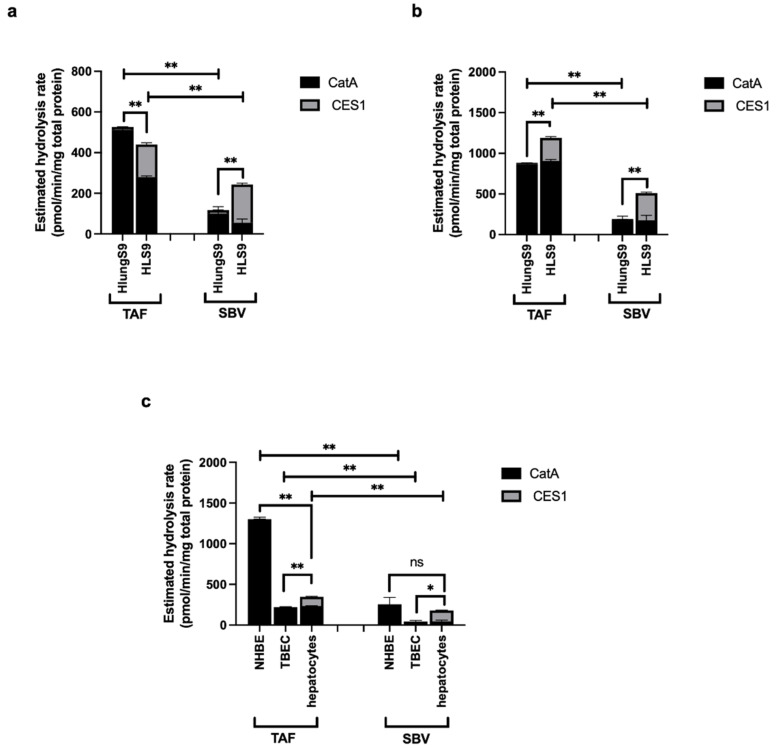
Estimated hydrolysis rates of TAF and SBV in human tissue S9 and primary cells. Estimated hydrolysis rates of TAF and SBV in human tissue S9 based on the enzyme abundance from the study by Li J. et al. [16] (**a**) and the study by Li R. et al. [13] (**b**) and in primary human cells (**c**). The estimated hydrolysis rate of a prodrug in a specific biological system was calculated by summing the CatA and CES1 mediated hydrolysis, which was calculated by timing the enzyme’s activity measured in this study (n = 3) with enzyme’s mean abundance extracted from previous studies (mean ± S.D.). NHBE: normal human bronchial epithelial cells; TBEC: tracheal bronchial epithelial cells. * *p* < 0.05 and ** *p* < 0.01 indicate statistically significant difference, “ns” indicates no significant difference. The difference between TAF and SBV hydrolysis rates in each specific biological system was analyzed by Student’s *t*-test; For each prodrug, the difference between HlungS9 and HLS9 was analyzed by Student’s *t*-test, and the differences among three primary cells were analyzed by ANOVA test.

The estimated hydrolysis rate of TAF and SBV in human tissue S9 and primary human cells are shown in Figure 7. For TAF, CatA was estimated to be the major contributor to its hydrolysis in HlungS9, HLS9, primary NHBE cells, TBEC cells, and primary human hepatocytes. While for SBV, CatA was predicted to be the major contributor in HlungS9, primary NHBE cells, and TBEC cells, CES1 was estimated to be the major enzyme hydrolyzing SBV in HLS9 and primary human hepatocytes. Overall, TAF was predicted to have a higher hydrolysis rate than SBV in human lung and liver tissue S9 and primary cells. In the human respiratory system (e.g., HlungS9, primary NHBE cells, and TBEC cells), the hydrolysis rate of TAF was estimated to be approximately 4-fold higher than that of SBV; in the human liver (HLS9 and primary human hepatocytes), TAF hydrolysis rate was predicted to be about 1-fold higher than SBV hydrolysis rate. Unlike TAF that was estimated to have a much higher hydrolysis rate in NHBE cells than in hepatocytes, the hydrolysis rates of SBV in NHBE cells and hepatocytes were expected to be comparable.

## 4. Discussion

Our ABPP study identified more than 20 active serine hydrolases in HlungS9. Among them, LYPLA1, CatA, and CES1 are likely to have the highest catalytic activity because they were enriched most quickly (within 1 min), and the enrichment folds were the highest. As a comparison, a few other serine hydrolyses, such as LYPLA2, CPVL, and SCPEP1, were enriched by the probe to a much less extent, and the enrichment required a longer period time of probe incubation. We then tested the activity of rhLYPLA1, rhCatA, and rhCES1 on hydrolyzing TAF. The results showed that TAF was not a substate of LYPLA1, whereas CatA and CES1 exhibited significant activity on TAF hydrolysis, an observation consistent with previous studies [10,11]. Moreover, CatA and CES1 were found to be involved in SBV hydrolysis. When evaluating an enzyme’s capability to metabolize its substrate drug in a specific tissue, both the abundance and catalytic activity of the enzyme should be taken into consideration. Unlike CatA, which is expressed in a broad range of tissues [16,30], CES1 is predominantly expressed in the liver [16,31]. In the context of COVID-19, human respiratory systems, including the airway and lung tissues, are the major infection sites [32], and thus are the most important target activating sites for antiviral prodrugs. It is noteworthy that, in human airway epithelial cells, the expression of CES1 was very low, while CatA expression was about 2-fold higher than CES1 [13,27]. CatA, therefore, appears to be a desirable enzyme for activating an easter prodrug in human respiratory system due to its high catalytic activity and considerable protein expression level in human lung tissue [13,16] and airway epithelial cells [13,27].

It has been indicated that the antiviral potency of TAF, SBV, and RDV was significantly associated with their hydrolysis rates in a cell, and the hydrolysis rate was highly dependent on the abundance of CatA and CES1 [12,13,14]. For example, a cell-dependent antiviral efficacy has been reported for SBV [33]. In that study, Huh-7 cells could generate much more TP-Nuc of SBV than A549 (416 vs. 36 pmol/10^6^ cells); consequently, SBV had stronger anti-Zika activity in Huh-7 cells than in A549 (IC_50_ 4 µM vs. >50 µM) [33]. The different antiviral effects can be explained by the much greater protein expression of CES1 in Huh-7 than in A549 [29].

CES1 is expressed in the cytoplasm and the endoplasmic reticulum lumen where pH is about 7.1–7.4, whereas CatA is mainly expressed in the lysosomes, which have an acidic interior pH of approximately 4.7 [34]. In this study, we used pH 5.5 and pH 7.5 to mimic the physiological environment for CatA and CES1, respectively, ensuring optimal reaction conditions for measuring CatA and CES1 activity in HlungS9 and HLS9. Both HlungS9 and HLS9 exhibited a relatively higher TAF hydrolysis activity at pH 5.5 than pH 7.5, suggesting a critical role of CatA in hydrolyzing TAF in both tissues. Our results demonstrated that CatA is the major enzyme responsible for activating TAF in the human lung. Based on the enzyme activity and abundance in HlungS9, CatA was estimated to account for approximately 97% of the total TAF hydrolysis in HlungS9. Our estimation of TAF hydrolysis in HlungS9 was consistent with the observations from the tissue S9 incubations (Figure 5) and the inhibitory study (Figure 6). It has been well documented that telaprevir does not affect CES1 activity, and BNPP does not hinder CatA activity [12,13]. We demonstrated that telaprevir could nearly abolish all TAF hydrolysis in HlungS9 at a concentration as low as 0.5 µM, while BNPP only showed a slight inhibitory effect at 50 µM. Previous studies showed that BNPP at 50 µM could eradicate CES1 activity [35,36]. Moreover, our results showed that the pH 5.5 incubation condition (optimal condition for CatA) could significantly enhance TAF hydrolysis in HlungS9, further suggesting that CatA might play a more important role in TAF hydrolysis in HlungS9 than CES1.

The analysis of published proteomics data revealed that the abundance ratios of CatA to CES1 in HlungS9 and primary human airway epithelial cells were much higher than that in HLS9 and primary human hepatocytes (Table 1). Compared with SBV, TAF had a significantly higher hydrolysis rate in HlungS9, which can be explained by its higher susceptivity to CatA relative to CES1. From a prodrug activation perspective, our results indicated that TAF could serve as an example for designing prodrugs with improved pulmonary hydrolysis. Our data also suggest that SBV was a less desirable candidate for COVID-19 treatment due to its slow activation in the human lung. Interestingly, the protein expression of CatA in lung tissues of COVID-19 patients was approximately 1-fold higher than non-COVID-19 patients, further indicating CatA could be a desirable target enzyme for activating anti-COVID-19 prodrugs. CatA expression in the liver is comparable to that in the lung, and an extensive TAF hydrolysis was also observed in HLS9. As such, instead of oral dosing, intravenous or inhalation administration should be adopted to avoid the extensive TAF metabolism in the liver.

Previous studies have shown that the susceptivity to CatA could be adjusted by modifying the ester moieties of ProTides using diverse amino acids and alcohols groups [14,37,38]. Recently, several nucleoside/nucleotide analogs have been identified as promising candidates against SARS-CoV-2, such as favipiravir [39] and molnupiravir (EIDD-2801) [40]. It is expected that the high susceptivity to CatA can be achieved by applying the ProTide technology to these nucleoside/nucleotide analogs using suitable ester moieties. Further investigations are warranted to evaluate whether a highly CatA susceptive ProTide form could improve the pulmonary activation of other nucleoside/nucleotide analogs.

Admittedly, this study has several limitations. First, we only used tissue S9 to profile the hydrolysis of TAF in the lung and liver. The tissue S9 fractions are usually isolated from a mixture of different types of cells in a tissue, and the abundance of enzymes in the tissue S9 may not reflect their intracellular expression in specific types of cells. Moreover, in contrast to intact cells, S9 fractions are incapable of evaluating the impact of drug transport across cell membranes on the activation of a prodrug. As such, primary cell culture experiments and in vivo studies are needed to verify the theory that high susceptivity to CatA can boost the ester prodrug activation in the lung.

## 5. Conclusions

TAF has a higher hydrolysis rate in HlungS9 and HLS9 than SBV. CatA is the major enzyme responsible for TAF hydrolysis in HlungS9. The abundance ratio of CatA to CES1 is much higher in primary human airway epithelial cells than in primary human hepatocytes, and the abundance ratio of CatA to CES1 is also higher in HlungS9 than in HLS9. The higher hydrolysis rate of TAF than SBV in HlungS9 may be attributed to the higher susceptivity of TAF to CatA.

## Figures and Tables

**Table 1:** Meta-analysis of the protein abundance of CES1 and CatA in human respiratory and hepatic tissues and cells.

Enzyme Resource	Study Reference	Sample Size	Assay Method and Quantification Type	Abundance		Abundance Ratio ^a^ CatA: CES1	CatA Contribution to Hydrolysis ^b^
				CatA	CES1		
Pooled HLungS9	[16]	4 donors, triplicate measurement	Label-free DIA MS-based proteomics, absolute quantification	0.130 ± 0.006 µg/mg total protein	1.702 ± 0.063 µg/mg total protein	1:13	97%
Pooled HLS9		200 donors, triplicate measurement		0.071 ± 0.004 µg/mg total protein	19.244 ± 0.083 µg/mg total protein	1:271	64%
HLung S9	[13]	n = 3	Western blot analysis	0.220 ± 0.004 µg/mg total protein	2.1 ± 0.9 µg/mg total protein	1:10	98%
HLS9		n = 3		0.23 ± 0.11 µg/mg total protein	34 ± 1 µg/mg total protein	1:148	76%
Primary human NHBE cells		n = 3		0.33 ± 0.10 µg/mg total protein	<0.1µg/mg total protein	>3.3:1	>99%
Primary human TBEC cells	[27]	n = 4	Label-free DDA MS-based proteomics, absolute quantification	0.056 ± 0.013 µg/mg total protein	0.019 ± 0.017 µg/mg total protein	3:1	>99%
Primary human hepatocytes	[28]	n = 7		0.059 ± 0.014 µg/mg total protein	13.7 ± 3.0 µg/mg total protein	1:232	67%
Lung tissue autopsy from COVID-19 patients	[26]	n = 30	Labeled DDA MS-based proteomics, relative quantification	1.501 ± 0.878	0.249 ± 0.069	-	-
Liver tissue autopsy from COVID-19 patients		n = 24		1.308 ± 0.300	3.465 ± 1.078	-	-
A549 cells (human pneumocyte type II carcinoma cells)	[29]	n = 3	Labeled DDA MS-based proteomics, relative quantification	0.765	0.797	-	-
Huh-7 cells (human hepatocellular carcinoma cells)		n = 3		1.11	13.96	-	-

**^a^** For relative abundance data, it is suitable to compare the abundance of same protein between cells/tissues, but not rational to compare different proteins in the same sample. **^b^** CatA contribution was calculated by dividing the CatA contribution by the summation of CatA and CES1 contrition. The enzyme contribution was estimated by timing the enzyme activity with its abundance. NHBE: normal human bronchial epithelial cells; TBEC: tracheal bronchial epithelial cells.

## Data Availability

Data are contained in the article and the Supplementary Materials.

## Supplementary Materials

The following are available online at https://www.mdpi.com/article/10.3390/pharmaceutics13101656/s1, Figure S1: Reaction of serine hydrolases with an ester substrate (a) and biotin activity-based probe (b) via nucleophilic attacking, Figure S2: rhLYPLA1 showed no activity for TAF hydrolysis, Table S1: Demographic information of the tissue S9 donors, Table S2: The gradient conditions of LC-MS/MS analysis, Table S3: Meta-analysis of the protein abundance of HINT1 in the human lung and liver.
