# Supplementary material for: Activation of Tenofovir Alafenamide and Sofosbuvir in the Human Lung and Its Implications in the Development of Nucleoside/Nucleotide Prodrugs for Treating SARS-CoV-2 Pulmonary Infection
Source: Pharmaceutics. 2021 Oct 11;13(10):1656. doi: 10.3390/pharmaceutics13101656 (PMC8540046; doi:10.3390/pharmaceutics13101656)
Supplement: Supplementary file 1 [file pharmaceutics-13-01656-s001.zip › pharmaceutics-1384423-supplementary.pdf]

pharmaceutics MDPI

Supplementary Materials: Activation of Tenofovir Alafenamide and Sofosbuvir in the Human Lung and Its Implications in the Development of Nucleoside/Nucleotide Prodrugs for Treating SARS-CoV-2 Pulmonary Infection

Jiapeng Li, Shuhan Liu, Jian Shi and Hao-Jie Zhu

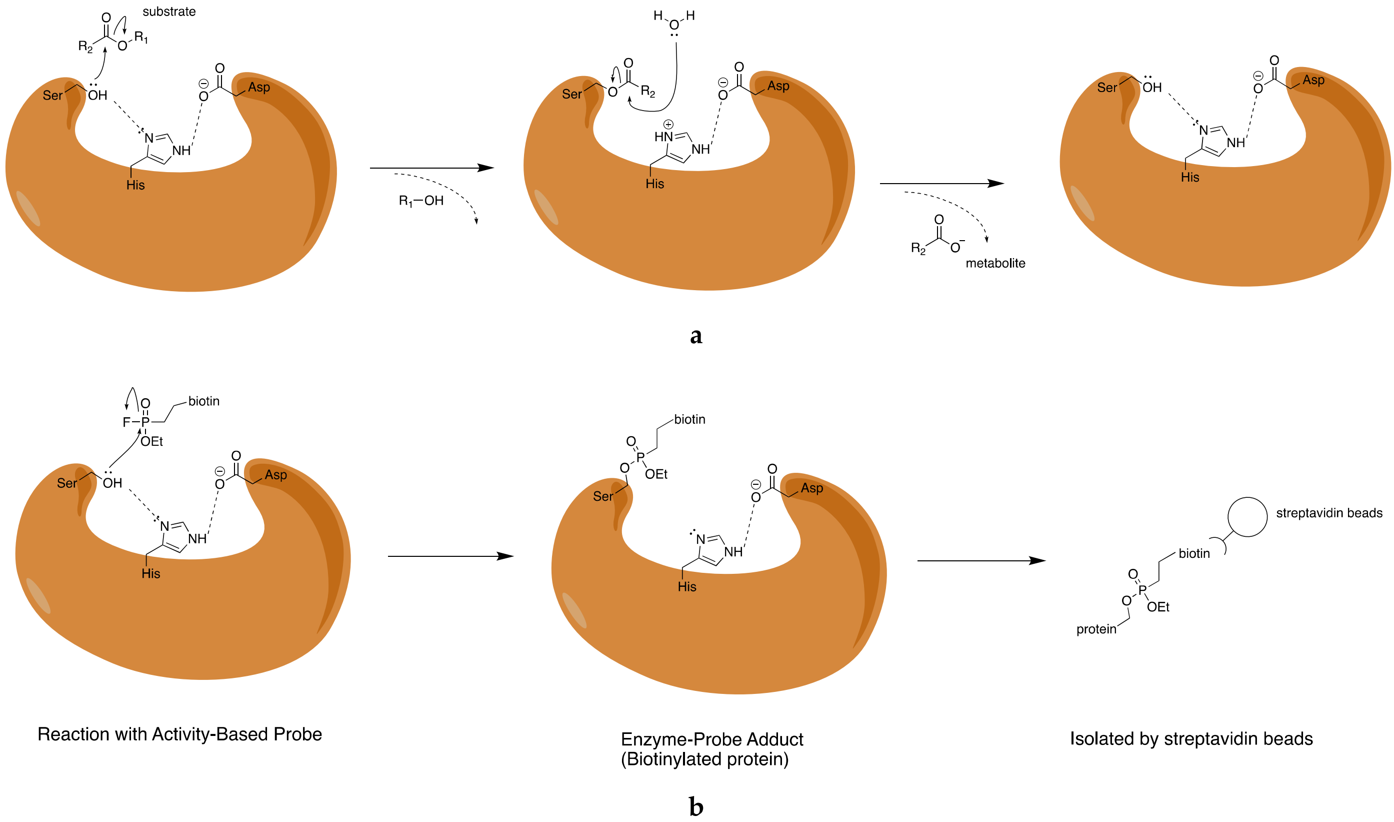

Figure S1. Reaction of serine hydrolases with an ester substrate (**a**) and biotin activity-based probe (**b**) via nucleophilic attacking [1].

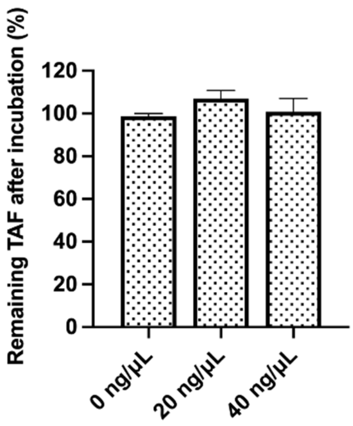

Figure S2. rhLYPLA1 showed no activity for TAF hydrolysis. 20 μM TAF was incubated with 20 and 40 ng/μL rhLYPLA1 in Tris pH 7.5 buffer at 37°C for 30 min. No significant reduction of TAF after incubations was identified.

Table S1. Demographic information of the tissue S9 donors.

Organ	Total sample number	Age (year)	Sex/ethnicity (number of samples)
Lung	4	22, 37, 49, 75	M/C (1); M/H (1); F/C (1); F/AA (1).
Liver	50	11-85	M/C (26); M/H (3); M/AA (1); F/C (19); F/A (1).

M = male; F = female. C = caucasian; H = hispanic; A = Asain; AA = African American.

Table S2. The gradient conditions of LC-MS/MS analysis.

Time (min)	A (%)	B (%)
0	97	3
68	70	30
73	60	40
75	20	80
78	20	80
79	97	3
90	end	end

Mobile phase: A: water containing 0.1% formic acid; B: acetonitrile containing 0.1% formic acid.

Table 3. Meta-analysis of the protein abundance of HINT1 in the human lung and liver.

Enzyme resource	Sample size	HINT1 Abundance[a]	Study reference
Pooled HLungS9	4 donors, triplicate measurement	0.165 ± 0.009 μg/mg total protein	[2]
Pooled HLS9	200 donors, triplicate measurement	0.588 ± 0.010 μg/mg total protein	
HLung S9	n = 3	2.4 ± 1.2 μg/mg total protein	[3]
HLS9	n = 3	4.5 ± 1.7 μg/mg total protein	
Primary human NHBE cells	n = 3	1.0 ± 0.3 μg/mg total protein	
Primary human TBEC cells	n = 4	0.802 ± 0.039 μg/mg total protein	[4]
Primary human hepatocytes	n = 7	0.50 ± 0.10 μg/mg total protein	[5]
Lung tissue autopsy from COVID-19 patients	n = 30	1.011 ± 0.721	[6]
Liver tissue autopsy from COVID-19 patients	n = 24	1.412 ± 0.328	
A549 cell line (lung cancer)	n = 3	0.919	[7]
Huh-7 cell line (liver cancer)	n = 3	1.14	

a: The unit (μg/mg total protein) is only applicable to absolute quantification data, is not applicable to the relative data obtained from the studies by Nie X. *et al.*[6] and Nusinow, D. P. *et al.* [7].

References

1. Tallman, K. R.; Levine, S. R.; Beatty, K. E., Small-Molecule Probes Reveal Esterases with Persistent Activity in Dormant and Reactivating Mycobacterium tuberculosis. *ACS Infectious Diseases* **2016,** 2, (12), 936-944.
2. Li, J.; Liu, S.; Shi, J.; Wang, X.; Xue, Y.; Zhu, H.-J., Tissue-Specific Proteomics Analysis of Anti-COVID-19 Nucleoside and Nucleotide Prodrug-Activating Enzymes Provides Insights into the Optimization of Prodrug Design and Pharmacotherapy Strategy. *ACS Pharmacology & Translational Science* **2021**, 4, (2), 870-887.
3. Li, R.; Liclican, A.; Xu, Y.; Pitts, J.; Niu, C.; Zhang, J.; Kim, C.; Zhao, X.; Soohoo, D.; Babusis, D.; Yue, Q.; Ma, B.; Murray Bernard, P.; Subramanian, R.; Xie, X.; Zou, J.; Bilello John, P.; Li, L.; Schultz Brian, E.; Sakowicz, R.; Smith Bill, J.; Shi, P.-Y.; Murakami, E.; Feng Joy, Y., Key Metabolic Enzymes Involved in Remdesivir Activation in Human Lung Cells. *Antimicrobial Agents and Chemotherapy* **2021,** 0, (ja), AAC.00602-21.
4. Foster, M. W.; Gwinn, W. M.; Kelly, F. L.; Brass, D. M.; Valente, A. M.; Moseley, M. A.; Thompson, J. W.; Morgan, D. L.; Palmer, S. M., Proteomic Analysis of Primary Human Airway Epithelial Cells Exposed to the Respiratory Toxicant Diacetyl. *Journal of Proteome Research* **2017,** 16, (2), 538-549.

5. Wiśniewski, J. R.; Vildhede, A.; Norén, A.; Artursson, P., In-depth quantitative analysis and comparison of the human hepatocyte and hepatoma cell line HepG2 proteomes. *Journal of Proteomics* **2016,** 136, 234-247.
6. Nie, X.; Qian, L.; Sun, R.; Huang, B.; Dong, X.; Xiao, Q.; Zhang, Q.; Lu, T.; Yue, L.; Chen, S.; Li, X.; Sun, Y.; Li, L.; Xu, L.; Li, Y.; Yang, M.; Xue, Z.; Liang, S.; Ding, X.; Yuan, C.; Peng, L.; Liu, W.; Yi, X.; Lyu, M.; Xiao, G.; Xu, X.; Ge, W.; He, J.; Fan, J.; Wu, J.; Luo, M.; Chang, X.; Pan, H.; Cai, X.; Zhou, J.; Yu, J.; Gao, H.; Xie, M.; Wang, S.; Ruan, G.; Chen, H.; Su, H.; Mei, H.; Luo, D.; Zhao, D.; Xu, F.; Li, Y.; Zhu, Y.; Xia, J.; Hu, Y.; Guo, T., Multi-organ proteomic landscape of COVID-19 autopsies. *Cell* **2021,** 184, (3), 775-791.e14.
7. Nusinow, D. P.; Szpyt, J.; Ghandi, M.; Rose, C. M.; McDonald, E. R.; Kalocsay, M.; Jané-Valbuena, J.; Gelfand, E.; Schweppe, D. K.; Jedrychowski, M.; Golji, J.; Porter, D. A.; Rejtar, T.; Wang, Y. K.; Kryukov, G. V.; Stegmeier, F.; Erickson, B. K.; Garraway, L. A.; Sellers, W. R.; Gygi, S. P., Quantitative Proteomics of the Cancer Cell Line Encyclopedia. *Cell* **2020,** 180, (2), 387-402.e16.
